# Antibacterial Activity of Epigallocatechin Gallate (EGCG) against *Shigella flexneri*

**DOI:** 10.3390/ijerph20064676

**Published:** 2023-03-07

**Authors:** Yini Zhang, Yeyue Zhang, Ruiqing Ma, Wanting Sun, Zheng Ji

**Affiliations:** 1 School of Geography and Tourism, Shaanxi Normal University, Xi’an 710119, China; 2 International Joint Research Centre of Shaanxi Province for Pollutants Exposure and Eco-Environmental Health, Xi’an 710119, China

**Keywords:** epigallocatechin gallate, biofilm, *Shigella flexneri*, inhibition mechanism, oxidative stress

## Abstract

*Shigella flexneri* (*S. flexneri*), a major intestinal pathogen, is a global public health concern. The biofilms formed by *S. flexneri* threaten environmental safety, since they could promote the danger of environmental contamination and strengthen the disease-causing properties of bacteria. Epigallocatechin gallate (EGCG) is an important catechin in tea, which has a high antibacterial activity. However, its antibacterial mechanism is still unclear. This research aims to quantify the antibacterial function and investigate the possible mechanism of EGCG inhibition of *S. flexneri*. The minimum inhibitory concentration (MIC) of EGCG against planktonic *S. flexneri* in the investigation was measured to be 400 μg/mL. Besides, SDS-PAGE and field emission scanning electron microscopy showed that EGCG interfered with protein synthesis and changed bacteria morphology. Through controlling the expression of the mdoH gene, EGCG was found to be able to prevent an *S. flexneri* biofilm extracellular polysaccharide from forming, according to experiments utilizing the real-time PCR test. Additional research revealed that EGCG might stimulate the response of *S. flexneri* to oxidative stress and prevent bacterial growth. These findings suggest that EGCG, a natural compound, may play a substantial role in *S. flexneri* growth inhibition.

## 1. Introduction

There are four species of *Shigella*: *Shigella dysenteriae* (*S. dysenteriae*), *Shigella boydii* (*S. boydii*), *Shigella flexneri* (*S. flexneri*), and *Shigella Sonnei* (*S. Sonnei*), which are intestinal invasive human pathogens that are Gram-negative, rod-shaped, non-spore-forming, non-motile, and facultatively anaerobic [1]. Shigellosis is caused by the infection of epithelial cells in the large intestine with *Shigella* bacteria. There is a risk of a lack of vaccine prevention or treatment, and antibiotic-resistant Shigellosis strains are on the rise [2]. According to reports, *Shigella* is the second most prevalent cause of global diarrheal fatalities and the most frequent cause of dysentery in underdeveloped nations, which contributes to an estimated 165 million people being infected and 1.1 million deaths annually, worldwide [3]. In China, shigellosis has been listed as the third most prevalent disease and has become the leading reason for illness-related deaths in children [4,5]. In Africa and Asia, the Global Enteric Multicenter Study examined the prevalence and cause of mild-to-severe diarrheal illness in children under the age of five and matched controls without diarrhea, and found that *S. flexneri* accounted for nearly 70% of Shigella case isolates, which was the most widespread isolated species in the world and particularly common in developing countries [6], and according to previous research, underdeveloped nations were more likely than industrialized nations to experience sickness and mortality due to *S. flexneri* in children under the age of five [7,8].

Shigella is typically present in aquaculture water and several other types of contaminated water, and as biological pollution, it may impair fetal development [9,10]. According to a prior study, increasing the lifespan of *S. flexneri* in aqueous conditions would result in a higher risk of infection transmission in water than *S. typhi* [11]. A preceding study described the spatiotemporal trends of *Shigella* incidence rates, and investigated complex risk modes that promote the spread of *Shigella* in the People’s Republic of China’s Jiangsu Province, through a geographic information system and autoregressive integrated moving average model, and the findings demonstrated that distances from roads, rivers, and lakes promoted the spread of *Shigella*, and put forward the significance of minimizing Shigella infections spread via water systems [12], so it is essential to remove *S. flexneri* in the process of water treatment.

In addition, *S. flexneri* is frequently found in food processing and wastewater treatment environments [13,14,15]. Free *S. flexneri* cells attached to solid surfaces to form microbial communities that were wrapped by aggregates of extracellular polymeric material, consisting of polysaccharides, proteins, DNA, and lipids, to form well-established *S. flexneri* biofilms, which provided a strong physical barrier to active cells and prevented them from a variety of environmental stresses. Bacteria in biofilms may be a thousand times better able to resist adverse environmental stress than the same species of planktic bacteria [16,17,18].

The most credible treatment for *S. flexneri* is antibiotics. The World Health Organization selected ciprofloxacin as the first choice for treatment in 2017 [19]. However, resistance of Shigella isolates to ciprofloxacin is becoming increasingly common. Combination therapy has been employed to treat shigellosis caused by ciprofloxacin-resistant *Shigella* isolates. A study investigated the antimicrobial activity of ciprofloxacin/phosphonomycin combinations against *S.flexneri* isolates, and found that the combination produced enhanced bacterial killing with phosphonomycin concentrations of 150 and 300 μg/mL, especially when combined with ciprofloxacin at 2.5 μg/mL [20]. However, an estimated 10 million people a year will die from multidrug resistant infections, by 2050, without interventions, more than from cancer and motor vehicle crashes combined, according to a report commissioned by the UK, and the global economic loss due to antimicrobial resistance is estimated at $100 trillion [21]. As pathogens evolve and mutate, multidrug resistance also contributes to Shigella infections. Scientists are gradually shifting the treatment of Shigella infections to other areas. In recent years, researchers have focused on the antimicrobial activity of natural plant extracts from a wide range of sources, because these natural substances are safe and environmentally friendly. For example, eugenol was confirmed to exhibit an antibacterial effect against *S. flexneri* [22].

The capacity of tea extract to prevent the survival of many food-borne viruses was shown to be quite important [23], it contains several tea polyphenols, including epigallocatechin (EGC), epigallocatechin-gallate (EGCG), epicatechin (EC), and epicatechin-gallate (ECG) [24]. EGCG has rich and diverse health benefits, antiviral, anticancer, and antioxidant effects are included. [25]. Despite the increasing interest in the applications of EGCG or tea extracts in human health, the influence of EGCG on the growth of *S. flexneri* has rarely been investigated. EGCG is the most abundant and antimicrobial active substance among tea polyphenols [26]. Some scholars have suggested that the main inhibition mechanism of Gram-positive bacteria with EGCG is that EGCG inhibits the formation and function of the bacterial cell wall, which is mainly due to the ability of EGCG to bind to peptidoglycan, the primary component of bacterial cell walls, and encourage peptidoglycan precipitation [27]. For Gram-negative bacteria such as *Pseudomonas aeruginosa* and *Escherichia coli* O157:H7, it was suspected that EGCG mainly kills bacteria by producing H_2_O_2_ and other reactive oxygen species [28]. However, the antibacterial mechanisms of other bacteria are unknown. The goal of this work was to look at EGCG’s potential mode of action against planktonic *S. flexneri* and its biofilm, as well as at EGCG’s antimicrobial efficacy.

## 2. Materials and Methods

### 2.1. Bacteria Strain and Antimicrobial Agents

Hope Biotechnology Company (Qingdao, China) provided the *S. flexneri* strain (ATCC 12022). Yuanye Biotechnology Company (Shanghai, China) provided the EGCG monomer (purity: 98%).

### 2.2. Detection of Antimicrobial Activity in Planktonic Conditions

#### 2.2.1. The Minimum Inhibitory Concentration Determination

The prepared EGCG stock solution was diluted with LB broth using the 2-fold method to final concentrations of 50, 100, 200, 400, and 800 μg/mL. *S. flexneri* was grown overnight in LB broth at 37 °C, 150 rpm for 12 h, and then the culture solution was diluted to an optical density at 595 nm (OD_595_) of 0.5 in sterile fresh LB broth, 50 μL of this was aspirated, distributed evenly among the 96 wells of a polystyrene microtiter plate, and cultivated for 24 h at 37 °C, with various doses of 50 μL EGCG. The OD_595_ measurement, with a spectrophotometer (Epoch, VT, USA), for all experimental groups determined the MIC [24].

#### 2.2.2. SDS-PAGE of Bacterial Proteins

The *S. flexneri* suspensions were grown in LB broth to an OD_595_ value of 0.5, and the *S. flexneri* cells were cultured with 50 and 400 μg/mL EGCG, respectively, while only sterile PBS solution without EGCG was added as a positive control. LB broth containing only EGCG solution without bacteria was the negative control, and both the experimental and control groups had a 12-hour incubation period in an oscillating incubator at 37 °C and 150 rpm. After the samples had been exposed to EGCG for 12 h, they were centrifuged at 8000× *g* for 5 min, with the supernatant being discarded. After cleaning, the *S. flexneri* cell precipitates were redissolved in sterile PBS. Before SDS-PAGE analysis, the bacterial cell solution was broken up by ultrasonication for 10 min (200 w), and then centrifuged for 3 min (8000 rpm). A BCA protein test kit (KeyGEN Biotechnology, Nanjing, China) was used for measuring the total protein content. After uniformly adjusting the protein quantities in both experimental and control groups, gels were prepared using the SmartBuffers^®^ any KD fastcast gels kit (1Use Biomedicine Co., LTD., Guangzhou, China), according to its instructions. After being dyed with Coomassie Brilliant Blue R250, and decolored with a solution made of glacial acetic acid, methanol, and distilled water, the protein bands could then be seen on the gels.

#### 2.2.3. Analysis Using a Field Emission Scanning Electron Microscope (FESEM)

The *S. flexneri* were cultivated at 37 °C for 6 h with EGCG at different doses (control, 50, 100, and 200 μg/mL), and then the cells were harvested by centrifugation at 8000 rpm for 3 min, before being cleaned three times with PBS. The samples were dehydrated in 25%, 50%, 75%, 95%, and 100% ethanol, before the washed cells were resuspended in 2.5% glutaraldehyde at 4 °C for 2 h. All samples were mounted on FESEM supports and vacuum-sputter-coated with gold, and examined microscopically on an FESEM (Nova NanoSEM 450, FEI, Brno, Czech) [29].

### 2.3. Detection of EGCG’s Impact on the Development of Biofilms

#### 2.3.1. Quantitative Evaluation of *S. flexneri* Crystal Violet Biofilm Development

After 12 hours of LB broth culture on *S. flexneri* cells, the optical density at 595 nm was 0.5, with a 1/2 LB dilution. A 96-well polystyrene microtiter plate was used to hold 200 μL of the *S. flexneri* solution, which was then grown at 37 °C for a day with various EGCG concentrations (control, 50, 100, and 200 μg/mL). The wells were lightly rinsed three times with PBS, to remove any leftover planktonic *S. flexneri* cells after the biofilm had formed. The biofilms were then fixed in 200 μL of 100% methanol for 15 min. After the methanol was removed, the biofilms were stained for 30 min with 0.1% crystal violet, rinsed with sterile PBS to remove any remaining dye, and then air-dried. The stained biofilms were dissolved in 95% ethanol for 12 h, and the optical density at Multiskan Spectrum (Thermo, Multiskan Go, Waltham, MA, USA) 570 nm was measured [30]. To several of the wells, 200 μg/mL of ciprofloxacin was added as an antibiotic control (CIP) [1].

#### 2.3.2. Determination of Viable Bacteria Inside and Outside *S. flexneri* Biofilm

The *S. flexneri* biofilm’s interior and exterior bacterium viability was determined using the plate counting method. The biofilm modeling is detailed in Section 2.3.1. After the incubation, the remaining bacterial solution was aspirated in the tube, avoiding scraping the inner wall of the tube, and 100 μL was taken on a sterilized plate, for counting. Then, 200 μL sterile PBS was added, the biofilm was evenly blown, and 100 μL of biofilm was sucked out and coated on the disinfection plate. After 24 h of culture at 37 °C, the cells were counted.

#### 2.3.3. RNA Extraction and Quantitative RT-PCR Evaluation

Following the methodology proposed by Kang et al. [1], transcript levels of the mdoH gene were quantified by qRT-PCR during the biofilm development of *S. flexneri* at various doses of EGCG. The gene-specific primers are shown in Table 1. The biofilm modeling is detailed in Section 2.3.1.

### 2.4. Antibacterial Mechanism

#### 2.4.1. Determination of the Effect of Antioxidants on EGCG

Antioxidants were added to the culture medium containing EGCG, and the inhibition mechanism of EGCG was initially determined by colony counting. Set up six experimental groups, as shown in Table 2.

The method for determination of the number of reculture colonies is as follows. The growth broth was injected with the 1% bacterial solution, which was then cultivated for 7 h at 37 °C and 180 rpm. The cultured bacterial suspension was then diluted to 10^−5^ using PBS. Then, 50 μL of each treated bacterial broth was coated on LB solid medium and incubated for 18 h at 37 °C. Colonies on each plate were counted after incubation.

#### 2.4.2. Determination of ROS, H_2_O_2_, SOD, and CAT

A PBS negative control was set up and incubated for two hours at 37 °C and 150 rpm, while the *S. flexneri* suspensions were cultured in LB broth until an OD_595_ value of 0.5 was reached. EGCG (1%) was then added to the bacterial suspension. After centrifuging the mixture at 8000 rpm for two minutes, the precipitate was retained and gently washed twice with PBS. Then, the bacterial solution was re-suspended by adding 5 mL of PBS. Using commercially available kits (Beyotime Institute of Biotechnology, Shanghai, China), ROS levels and hydrogen peroxide concentrations were assessed. SOD activity was assessed using the total SOD assay kit with WST-8 (Beyotime Institute of Biotechnology, Shanghai, China), along with the BCA protein assay kit (Beyotime Institute of Biotechnology, Shanghai, China), while CAT activity was measured with the BCA protein assay kit (Beyotime Institute of Biotechnology, Shanghai, China) and catalase (CAT) assay kit (Nanjing Jiancheng Bioengineering Institute, Nanjing, China). Finally, the SOD and CAT activities were computed following the guidelines provided in the kits.

### 2.5. Analytical Statistics

To evaluate statistical differences, SPSS 23.0 (SPSS, New York, NY, USA) was utilized. *p* < 0.01 was regarded as extremely significant, while *p* < 0.05 was considered statistically significant. In this study, each experiment was run in triplicate.

## 3. Results

### 3.1. The Antibacterial Activity of EGCG against a Strain of S. flexneri

**Figure 1:**
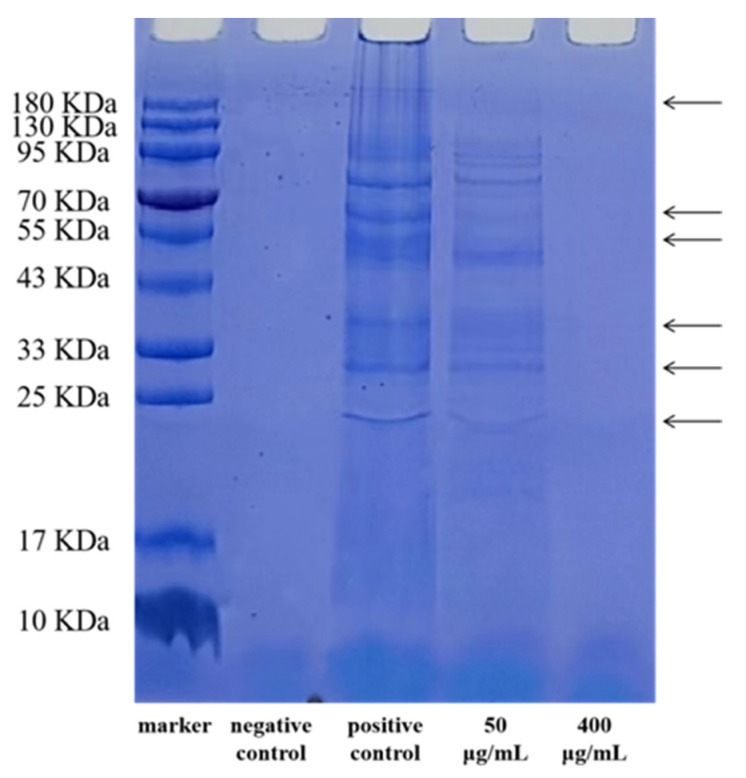
SDS-PAGE analysis of the impact of EGCG on intracellular proteins of *S. flexneri*.

**Figure 2:**
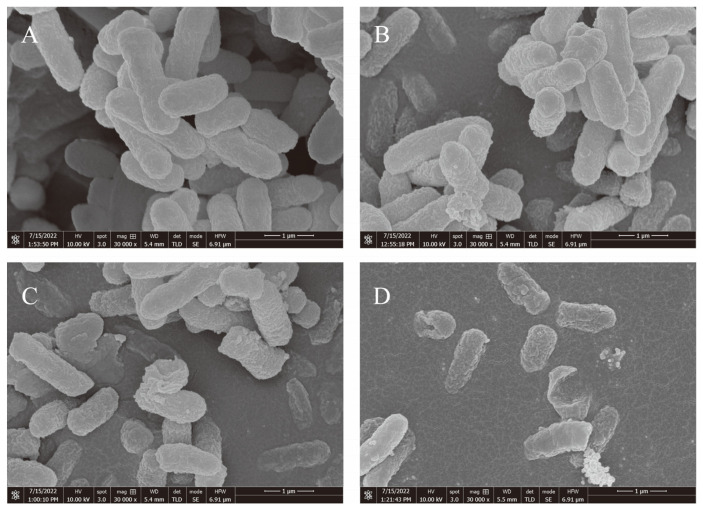
Using FESEM, the morphology of *S. flexneri* cells was examined. (**A**) Control; *S. flexneri* cells given EGCG at (**B**) 50 μg/mL; (**C**) 100 μg/mL; and (**D**) 200 μg/mL.

Table 3 and Table 4 show that EGCG’s MIC for *S. flexneri* was 400 μg/mL. The SDS-PAGE patterns of the proteins from *S. flexneri* cell proteins are shown in Figure 1. Compared with the positive control group, the bands in the 50 μg/mL EGCG-treated group were absent or weakened at the arrowed points in Figure 1, which proves that the protein synthesis of the *S. flexneri* was inhibited, while the protein bands in the 400 μg/mL EGCG-treated group were utterly absent, which proves that the *S. flexneri* might have died. Thus, EGCG might interfere with the production of proteins in bacterial cells. The microstructures of *S. flexneri* cells treated with EGCG by FESEM, revealed that EGCG gave rise to significant morphological alterations in *S. flexneri* cells. As can be seen from Figure 2, *S. flexneri* cells that were untreated had a typical Gram-negative bacillary structure, with a perfect and undamaged look (Figure 2A). When the cells were exposed to EGCG at 50 and 100 μg/mL, the cell morphology was disrupted, and cell components were exuded (Figure 2B,C). The cells given 200 μg/mL of EGCG showed almost no normal morphology (Figure 2D). This indicated that EGCG combined with and penetrated the bacterial cell surface and inhibited the growth of bacteria. Therefore, *S. flexneri* growth could be inhibited by EGCG, and as the EGCG concentration was increased, so was the inhibitory effect of EGCG on *S. flexneri* development.

### 3.2. EGCG Inhibits the Formation of Biofilms

**Figure 3:**
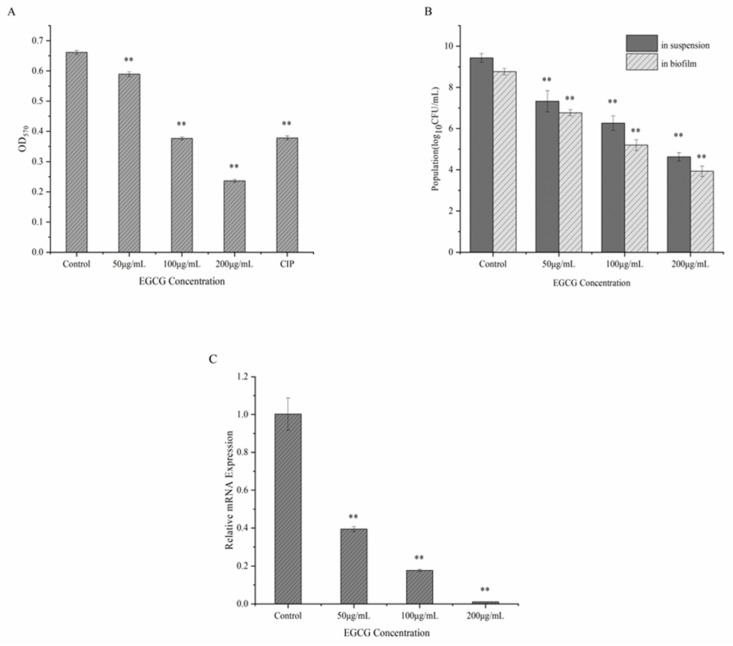
(**A**) Effect of EGCG on developing *S. flexneri* biofilms. (**B**) Effect of various EGCG treatment concentrations on the number of live bacteria in suspension and in the *S. flexneri* biofilm. (**C**) Expression of mdoH by different concentrations of EGCG treatment. Each bar represents the mean ± SD of three independent experiments, ** *p* < 0.01 versus with the control.

The influence of various concentrations of EGCG on *S. flexneri* biofilms were measured by crystal violet assays. A dose of 50 μg/mL of EGCG was shown to be able to prevent the development of biofilms (Figure 3A). Following incubation with 200 g/mL of EGCG, the biofilm inhibition rate was 64.28%. The increase in EGCG concentrations enhanced the inhibition of *S. flexneri* biofilm formation. From Figure 3B, EGCG concentration may have had an effect on bacteria both within and outside of the *S. flexneri* biofilm that was dose-dependent, suggesting that EGCG may pierce the biofilm of *S. flexneri* and suppress bacterial activity in the membrane. qRT-PCR was used to identify the expression of the mdoH gene following EGCG administration. The *S. flexneri* cells showed a restrictive impact on mdoH gene transcription during biofilm formation, when exposed to various doses of EGCG. (Figure 3C). With the increasing concentration of EGCG, the expression of the mdoH gene was gradually suppressed, which might indicate that the transcriptional increase in the mdoH gene was prevented by EGCG treatment. Thus, it was demonstrated that EGCG effectively prevented *S. flexneri* from forming biofilms.

### 3.3. Possible Mechanism of EGCG against S. flexneri

**Figure 4:**
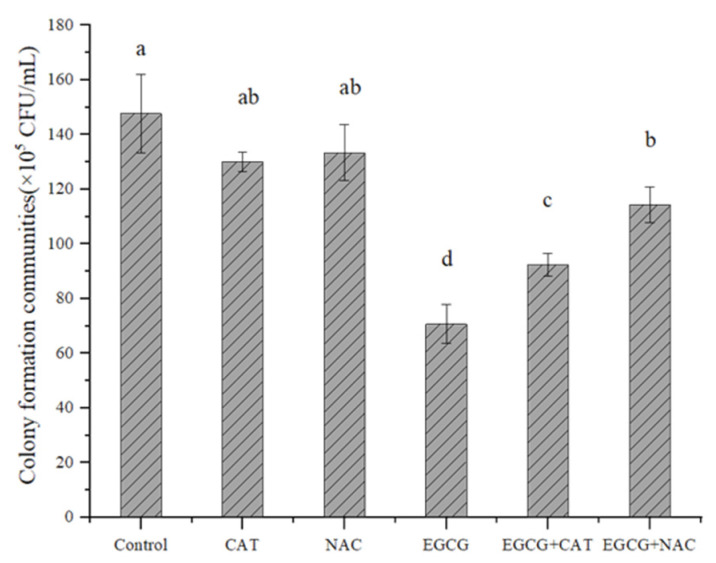
Effect of antioxidants on the subculture C.F.U of *S. flexneri* treated with EGCG. Means presented for a parameter that are followed by the same letter (for the same filling pattern) were not significantly different at a 5% confidence interval.

**Figure 5:**
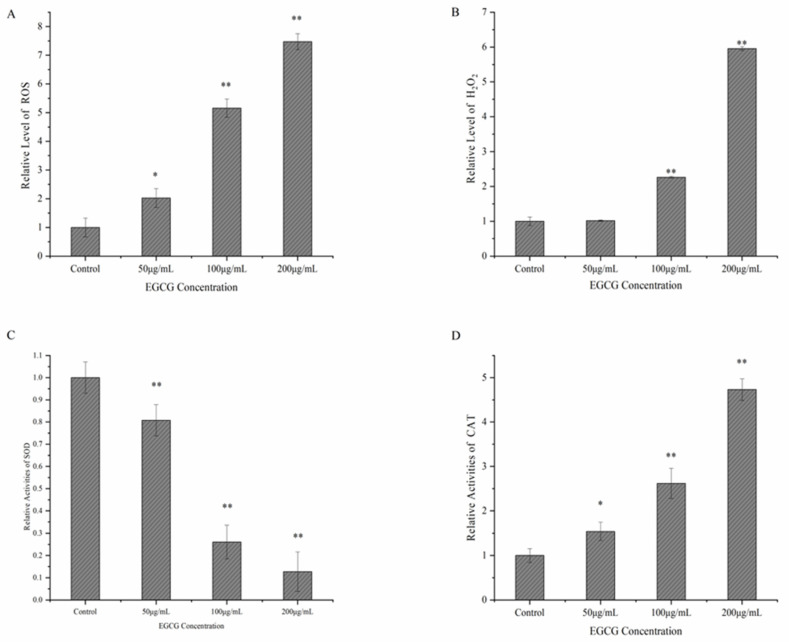
(**A**) *S. flexneri* treated with EGCG had a rather high level of intracellular ROS. (**B**) *S. flexneri* intracellular H_2_O_2_ relative level after EGCG treatment. (**C**) Relative activities of intracellular SOD. (**D**) Relative activities of intracellular CAT. Each bar represents the mean ± SD of three independent experiments, * *p* < 0.05 and ** *p* < 0.01 versus with the control.

As shown in Figure 4, the number of colonies was found to be 52.14%, 45.60%, and 14.45% lower in the EGCG group, EGCG + CAT group, and EGCG + NAC group than in the control group, respectively. Compared with the EGCG group, the number of colonies in the EGCG + NAC group increased by 44.06%, and there was no remarkable variation in the colony number of the EGCG + CAT group. This showed that NAC could remarkably suppress the antibacterial activity of EGCG, while CAT played a minor role in the antibacterial activity of EGCG (Figure 4). In this work, the generation of intracellular reactive oxygen species (ROS) and intracellular H_2_O_2_, in the bacterial cells treated with EGCG, increased noticeably (Figure 5A,B). Then, the SOD and CAT activity of *S. flexneri* exposed to EGCG were assessed. The findings showed that EGCG decreased the *S. flexneri* strains’ relative SOD activity (Figure 5C,D).

## 4. Discussion

The emergence of germs that are resistant to antibiotics poses a serious threat to public health, since it prevents the use of earlier, conventional treatments and increases the likelihood of treatment failure. Shigella, the major disease-causing organism of Shigellosis, have developed stronger resistance against more and more antibiotics, and shigellosis outbreaks are mainly caused by drug-resistant strains [31]. Previous investigations have revealed that *S. flexneri* has an above-average resistance to tetracycline, ampicillin, nalidixic Acid, and cotrimoxazole, while the pooled average resistance to chloramphenicol was more than 50% [32]. Therefore, it is necessary to further study and explore drugs to which *S. flexneri* show lower resistance. Polyphenolic substances have been shown to suppress the growth of numerous bacteria, in addition to being advantageous for human health [33]. Recently, EGCG has been shown to be a good antimicrobial agent against both of the well-known pathogens of the oral cavity, known as *Streptococcus mutans*, the main pathogen of tooth decay, as well as *Porphyromonas gingivalis*, contributing to periodontal disease [34,35].Thus, this study assessed the inhibitory effect of EGCG on *S. flexneri.*

It was found that the MIC of EGCG against *S. flexneri* was lower than most of the other antibacterial substances, for example, gallic acid’s MIC value against planktonic *S. flexneri* cells was 2 mg/mL, while the MIC value of polyphenolic extracts of edible flowers of Sesbania grandiflora was 0.028 mg/mL against planktonic *S. flexneri* [1,36]. In previous research, an aqueous ethanol extract of Euphorbia prostrata was tested for its ability to suppress *S. dysenteriae* type 1 bacterial activity, both in vitro and in vivo, and it was found to be effective against *Shigella* isolates with MICs of 3500–12,000 μg/mL [37]. NPs commonly cause harm to bacterial targets, destroy membrane-loaded cells and their integrity, and release free oxygen radicals. Shigella has recently been successfully treated with copper oxide nanoparticles, and when *S. sonnei* was treated with 33 nm NPs, the MIC of copper oxide nanoparticles reached 2500 μg/mL [38]. As shown above, the current MIC of most inhibitor alternatives is high. Further, by SDS-PAGE, EGCG was found to reduce bacterial protein synthesis, and this finding corresponds with a previous study, showing that EGCG can inhibit the synthesis of *Staphylococcus aureus* cellular proteins [39]. Moreover, this study examined the microstructure of *S. flexneri* cells exposed to EGCG by FESEM, and we found that EGCG leads to visible morphological changes in the *S. flexneri* cells, as shown in Figure 2. By determining these in the above experiments, the inhibitory effect of EGCG on *S. flexneri* was first demonstrated in this work.

Most studies have shown that EGCG achieves its antibacterial purpose by stimulating oxidative stress [28,40]. In previous studies, it has been demonstrated that the formation of hydroxyl radicals mediates the antibacterial action of EGCG against *Enterococcus faecalis* [41]. Joshua et al. [42] found that EGCG can generate H_2_O_2_ by oxidation in the cell culture medium. So, treating *S. flexneri* with EGCG might have increased the generation of H_2_O_2_ and intracellular ROS, and increased the activity of CAT, for clearing the excess H_2_O_2_. Studies have shown that decreased levels of the SOD enzyme lead to an increase in the level of intracellular ROS, which in turn leads to the inhibition of bacterial activity. When Li et al. [43] assessed the inhibitory effect of EGCG on *Vibrio mimicus*, they found that it reduced the activity of the SOD enzyme, and led to an increase in the intracellular ROS level of *Vibrio mimicus*. Meanwhile, Bai et al. [22] obtained similar results when they studied the inhibitory effect of eugenol on *S. flexneri*. Previous studies have shown that the excessive accumulation of ROS promotes oxidative damage to cell membranes and destroys cell membrane integrity [44]. This was the same as the results of this study. Therefore, it was proposed that EGCG may prevent *S. flexneri* from acting by producing hydroxyl radicals.

A persistent biofilm generated by *S. flexneri* would cause contamination in the manufacturing, storage, and sale processes [1]. Planktonic bacteria are easier to clean and remove than bacteria in biofilms [16,45]. Most of the current studies have focused on Gram-positive bacteria. For *Enterococcus faecalis*, the biofilm could be completely eradicated using 500 μg/mL EGCG, while 100 μg/mL EGCG could significantly reduce *L. monocytogenes* ATCC 19114 biofilm formation, when compared to the control [24,41]. The quantitative analysis of crystal violet in this study, showed that 50 μg/mL EGCG, present for 24 h at 37 °C, has an inhibitory impact on *S. flexneri* biofilm formation, considerably reducing the amount of *S. flexneri* biofilm that formed. By using a plate counting approach at the MIC level, it was possible to identify the effect of EGCG on living cells in a mature biofilm, and it was discovered that EGCG is capable of killing the living bacteria in the biofilm. Therefore, EGCG was capable of eradicating the biofilm formation of *S. flexneri*. Combined with the previous results of the MIC, SDS-PAGE, and FESEM experiments (shown in Table 1, Figure 1 and Figure 2), EGCG was shown to be effective in killing the cells of live *S. flexneri*, leading to the inference that EGCG might be effective in preventing the transmission of Shigellosis.

According to one previous study, gallic acid treatment lowered the mdoH gene’s expression in *S. flexneri* biofilm cells, and decreased the biofilm’s polysaccharide content [1]. Another study found that the presence of 62.5 μg/mL of EGCG significantly reduced the formation of extracellular polymeric compounds in *S. mutans* biofilms, whereas the concentration of 15.6 μg/mL of EGCG was sufficient to suppress GTF expression in *S. mutans* [46]. The OpgH protein, which is necessary for the assembly of the poly-glucose backbone, encoded by the mdoH gene, was critical for glucosyltransferase (GTF) activity, essential in the synthesis of exopolysaccharides [47]. Simultaneously, the secretion of exopolysaccharides plays an essential role in the development of biofilms [16,48]. Thus, the current study assessed how EGCG affected the growth of *S. flexneri* biofilms by altering the expression of the mdoH gene. This finding showed that EGCG blocked the mdoH gene’s expression, which in turn prevented the production of polysaccharides. Our finding corresponds with the previous studies, showing that, the effective inhibition of mdoH gene expression could disturb polysaccharide synthesis and thus prevent the *S. flexneri* biofilm formation.

In recent studies, it was discovered that the currently popular approach of using tea polyphenols as an adjunct disinfectant for ozone and ultraviolet light, etc., had a good disinfecting effect. Tea polyphenols are a natural and eco-friendly plant preparation, a renewable green resource, and they exhibit good oxidative and bactericidal properties, so they show a great potential to replace traditional disinfectants [49]. In previous research, results have shown that when tea polyphenols were used as a disinfectant for drinking water treatment, and the dosage was greater than 0.1 g/L for 20 min, the effluent from the filter tank might fulfill drinking water quality requirements (CJ/T 206-2005), and it showed good disinfection persistence over a period of two days, but a higher dosage of tea polyphenols will result in increased economic costs and higher effluent color, limiting the application possibilities [50]. However, taking the combined disinfection of tea polyphenols and ozone as an example, according to a previous study, the best disinfection effect of the combined disinfectant was found when the ozone dosage was 2.5 mg/L, the ozone contact time was 25 min, and the tea polyphenols dosage was 20 mg/L. This will ensure the effluent’s safety from microbes under these circumstances [49]. Another study found that combined disinfection by tea polyphenols and ozone resulted in an average clearance rate of 56.5% of more than 20 antibiotic resistance genes with high water content, including MacB. Tetracyclines, sulfonamides, -lactams, aminoglycosides, and other resistance genes were also effectively removed by the combined disinfection, filling the gap left by ozone disinfection’s inability to remove tetracycline and sulfonamide resistance genes completely. It is possible that the efficient removal of Gram-negative bacteria, which serve as the primary host cells for tetracycline resistance genes, by the combined disinfectants [51]. EGCG, the most abundant and active catechin, is also the main component of tea polyphenols with an antibacterial effect [52]. Thus, EGCG might serve as an adjunct disinfectant for other disinfection methods, and it is important to study the mechanism of EGCG inhibition for its better development in the field of disinfection.

## 5. Conclusions

During the present investigation, EGCG exhibited an inhibitory effect on planktonic *S. flexneri*. Meanwhile, EGCG was shown to hinder the production of an *S. flexneri* biofilm and suppress bacteria within the biofilm. The expression of the mdoH gene was downregulated in the EGCG-treated biofilm cells. The principal way that EGCG prevents the growth of the *S. flexneri* biofilm is by preventing the production of extracellular polysaccharides, which make up the majority of the biofilm. Besides, EGCG might activate the oxidative stress response of *S. flexneri*, which results in the inactivation of *S. flexneri*. Our research offers fresh perspectives on the potential of EGCG as a natural, safe polyphenol that may prevent the growth of *S. flexneri* biofilms and lessen the risk of Shigella infections. It also raises the prospect of EGCG’s future use in the disinfection of drinking water and in reducing microbial pollution.

## Figures and Tables

**Table 1:** Real-time PCR was performed using the primers.

Gene	Nucleotide Sequence of Primers (5′-3′)	Amplicon Size (bp)
mdoH	5-TACCATCCGCCGTTACATTC-3	130
	5-ATCCTGACCAACCATATCCATAG-3	
16S rRNA	5-GGGACCCGCACAAGCGGTGG-3	191
	5-GGGTTGCGCTCGTTGCGGGA-3	

**Table 2:** The six experiment groups.

Reagent Groups	1	2	3	4	5	6
EGCG	-	200 μg/mL	-	-	200 μg/mL	200 μg/mL
CAT	-	-	5 μg/mL	-	5 μg/mL	-
NAC	-	-	-	16.2 μg/mL	-	16.2 μg/mL

Note: “-” means, did not add this reagent.

**Table 3:** The MIC of EGCG for *S. flexneri*.

Strain	EGCG Concentration (μg/mL)					
	0	50	100	200	400	800
*S. flexneri*	+	+	+	+	-	-

Note: “+” means more bacteria, “-” means no visible bacteria.

**Table 4:** The determination results of OD_595_ by spectrophotometer.

Initial Concentration of EGCG (μg/mL)	OD_595_ of EGCG	OD_595_ of Bacterial Solution after Inhibition	OD_595_ Difference (OD_595_ of Bacterial Solution after Inhibition—OD_595_ of EGCG)
0	0.069	0.688	0.619
50	0.158	0.564	0.406
100	0.168	0.497	0.329
200	0.204	0.449	0.245
400	0.331	0.398	0.067
800	0.586	0.837	0.251

## Data Availability

Not applicable.
